# γ-secretase inhibitors, DAPT and RO4929097, promote the migration of Human Glioma Cells via Smad5-downregulated E-cadherin Expression

**DOI:** 10.7150/ijms.50484

**Published:** 2021-04-26

**Authors:** Shun-Fu Chang, Wei-Hsun Yang, Chun-Yu Cheng, Sheng-Jie Luo, Ting-Chung Wang

**Affiliations:** 1 Department of Medical Research and Development, Chang Gung Memorial Hospital Chiayi Branch, Chiayi, Taiwan.; 2 Department of Neurosurgery, Chang Gung Memorial Hospital, Chiayi, Taiwan.; 3 Graduate Institute of Clinical Medical Sciences, College of Medicine, Chang Gung University, Taoyuan, Taiwan.; 4 Department of Biomedical Sciences and Institute of Molecular Biology, National Chung Cheng University, Chiayi, Taiwan.; 5 College of Medicine, Chang Gung University, Taoyuan, Taiwan.

**Keywords:** bone morphogenetic proteins, E-cadherin, γ-secretase, glioma, Smad1/5

## Abstract

Malignant gliomas are a type of central nervous system cancer with extremely high mortality rates in humans. γ-secretase has been becoming a potential target for cancer therapy, including glioma, because of the involvement of its enzymatic activity in regulating the proliferation and metastasis of cancer cells. In this study, we attempted to determine whether γ-secretase activity regulates E-cadherin to affect glioma cell migration. The human glioma cell lines, including LN18 and LN229, and the γ-secretase inhibitors, including N-[N-(3,5-difluorophenacetyl)-L-alanyl]-S-phenylglycine t-butyl ester (DAPT) and RO4929097, were used in this study. It was shown that γ-secretase activity inhibition by DAPT and RO4929097 could promote LN18 and LN229 glioma cell migration via downregulating E-cadherin mRNA and protein expressions, but not via affecting E-cadherin protein processing. In addition, γ-secretase activity inhibition was regulated by bone morphogenetic proteins-independent Smad5 activation in glioma cells. Moreover, endogenous Smad1 in glioma cells was found to play an important role in regulating E-cadherin expression and subsequent cell migration but did not affect DAPT-stimulated effects. These results help further elucidate the molecular mechanisms of γ-secretase activity regulation involved in controlling glioma cell malignancy. Information about a potential role for Smad1/5 activity upregulation and subsequent E-cadherin downregulation during inhibition of γ-secretase activity in the development of gliomas is therefore relevant for future research.

## Introduction

Malignant gliomas are one of the most prevalent type of central nervous system cancer in adult humans. Although the molecular mechanisms underlying malignant glioma function have been extensively investigated and current clinical treatments are multidisciplinary, including surgical resectioning, chemotherapy, and radiotherapy, the median survival rates and survival outcomes are still low and are not improved in the past several decades 1-2. The most prominent reason underlying this phenomenon is the high invasiveness of malignant gliomas, with clinical observations during early diagnoses typically noting the infiltration of surrounding brain tissue by glioma cells 3-4. Moreover, the genotypic and phenotypic heterogeneities within glioma tumor masses and stem cell-like characteristics of the glioma cells contribute to their aggressive invasiveness, infiltration, and migration. Such heterogeneity can also lead to the development of drug resistance 5-10. A more detailed investigation of the mechanisms underlying the invasive behavior of gliomas is thus still an urgent and important issue to investigate in order to improve glioma-targeting therapies.

γ-secretases are multisubunit protein complexes that cleave type I transmembrane proteins, including p75, Notch, and N-/E-cadherin 11-12. To date, over 100 substrates for γ-secretases have been found and, notably, most of these substrates play important but distinct functions in developing and adult tissues. It has therefore been suggested that the regulation of γ-secretase activity may control a large variety of cellular events 11-12. γ-secretase has been an increasingly relevent potential therapeutic target since its role in Alzheimer's disease has been identified. In addition, cleavage of multiple substrates by γ-secretase has been implicated in cancer development. For example, p75 cleavage is linked to gliomas, Notch cleavage is connected to many types of cancers (including breast and non-small-cell lung cancer), and E-cadherin cleavage is associated with colorectal cancer 1-2, 15-19. A number of γ-secretase inhibitors for clinical application have thus been developed. However, ensuring the specificity of these therapeutic γ-secretase inhibitors is difficult because of the wide variety of γ-secretase substrates, which are involved in many different signaling pathways and cellular events. The role of γ-secretases in gliomas is clinically relevant because accumulating data indicate that many γ-secretase substrates, including p75, Notch, and N-/E-cadherin, may regulate glioma pathogenesis and malignancy 15, 20-21. Thus, additional molecular understanding of how γ-secretases regulate glioma development is critical for developing future treatment strategies.

Bone morphogenetic proteins (BMPs), which are originally identified as bone-growth inducers, have been indicated as context-dependent stimulators now because of their multifunctional roles in physiological and pathophysiological processes in multiple tissue types. BMPs bind to their specific membrane receptors, activate Smad1/5 signaling and then regulate the target genes transcriptions 22. BMP signaling has also been found to act as either an inducer or repressor in different types of cancers 23-24. Moreover, in addition to playing a major role in neural development, recently, BMP signaling has also been linked to malignant glioma 22, 25-26. However, their precise role and mechanism are still unclear. BMP signaling and Notch/cadherin (γ-secretases substrates) signaling have been proposed to have important related functions in various cell types 27-28. Therefore, in this study, we investigated the functional and regulatory relationships between γ-secretases and BMP signaling in glioma cells.

N-[N-(3,5-difluorophenacetyl)-L-alanyl]-S-phenylglycine t-butyl ester (DAPT) and RO4929097 are two of the most widely used γ-secretase inhibitors 18, 29. In the present study, we investigate whether inhibition of γ-secretases activity by DAPT and RO4929097 decreases N-/E-cadherin protein processing, subsequently inhibiting glioma cell migration. However, it was surprisingly found that DAPT and RO4929097 unexpectedly inhibited E-cadherin (not N--cadherin) mRNA/protein expression and consequently promoted glioma cell migration via BMP-independent Smad5 activation. Our findings elucidate a possible role and mechanism for γ-secretase activity inhibition in regulating E-cadherin transcription and glioma cell migration, which may be relevant for future development of drugs targeting malignant gliomas.

## Methods

### Materials

DAPT was purchased from Sigma (St Louis, MO, USA). RO4929097 was purchased from Cayman Chemical (Ann Arbor, MI, USA). Noggin was purchased from BioLegend (San Diego, USA). All other chemicals of reagent grade were obtained from Sigma (St Louis, MO, USA).

### Cell culture

LN18 and LN229 human glioma cell lines were obtained from the American Type Culture Collection (Manassas, VA, USA). The cells were cultured as a monolayer in Dulbecco's modified Eagle's medium (Gibco, Grand Island, NY, USA) supplemented with 10% fetal bovine serum (Gibco, Grand Island, NY, USA) and 1% Penicillin-Streptomycin (Gibco, Grand Island, NY, USA) and maintained at 37 °C in a humidified 5% CO_2_ incubator.

### Quantitative real-time PCR

Total cellular RNA was extracted using an RNeasy Mini Kit (Qiagen Ltd, QIAGEN #74104) following the manufacturer's instructions. A total volume of 20 μl of cDNA was then prepared by reverse transcribing 2 μg of total cellular RNA using the SuperScript IV First-Strand Synthesis System (Invitrogen #18091050) using 20-mer oligo (dT) primers. Quantitative real-time PCR was then performed using a Quantinova SYBR GREEN PCR Kit (QIAGEN #208154). β-actin mRNA expression was used as an internal control. The sequences of oligonucleotide primers used for human E-cadherin and β-actin amplification were as follows: E-cadherin: 5'-TGCTCACATTTCCCAACTCC-3' and 5'-TTGCCTTCTTTCTCTTTGTT-3'; β-actin: 5'-ATGATATCGCCGCGCTCGT-3' and 5'-CGCTCGGTGAGGATCTTCA-3'. Each experiment was done in triplicate at minimum and the results are representative of at least three separate experiments.

### Western blot analysis

Western blotting was performed as previously described. Quantified cell lysates were suspended in cell lysis buffer (20 mM Tris-HCL pH 7.5, 1% Triton X-100, 10 mM EDTA, 1 mM EGTA, 2 mM sodium orthovanadate, and protease inhibitor cocktail (Roche, Indianapolis, IN, USA)). After centrifugation at 12000 rpm and 4 °C, proteins were resolved on a 7% SDS-PAGE gel then transferred onto a PVDF membrane (Millipore, Billerica, MA, USA). The membrane was then blocked with 8% non-fat milk for 2 hours at room temperature and probed with primary antibodies (1:1000 dilution) at 4 °C overnight. The primary antibodies used in this study were: anti-E-cadherin (BD610181) and anti-N-cadherin (BD610920)(BD Biosciences, San Jose, California, USA), anti-p-Smad1/5/9 (#13820; Cell Signaling Technology, Beverly, MA, USA), anti-Smad1/5/9 (sc-6031-R; Santa Cruz, CA, USA), and anti-β-actin (A5441; Sigma-Aldrich). Membrane was then rinsed with TBST and incubated with horseradish peroxidase-conjugated secondary antibody for 1 h at room temperature. Bands were visualized using a SuperSignal West Femto Maximum Sensitivity Substrate kit (Pierce, Waltham, MA, USA) and quantified using ImageJ software. The protein expression and phosphorylation levels were nomalized with the β-actin and total proteins, respectively, and the DAPT-/RO4929097-treated groups were compared to the untreated control group.

### Cell fractionation

The cytosolic, membrane and nuclear proteins were isolated and purified according to the protocols of the FractionPREP cell isolation kit (BioVision, Milpitas, California, USA). Fractionation results were further confirmed by Western blot with specific antibodies for GAPDH (a cytosol marker), Na^+^/K^+^-ATPase (a membrane marker) and YY1 (a nuclear marker).

### Migration (transwell) assay

Migration assays were performed in Boyden chambers using polyethylene terephthalate membranes with 8 μm pores with Falcon cell-culture inserts (BD Biosciences, Bedford, MA) according to the manufacturer's protocols. In brief, cells (5×10^4^) were seeded on the upper wells and allowed to migrate through the membrane at 37 °C in humidified air with 5% CO_2_. After 24 h, the filter membrane between the top and bottom chambers was removed. The cells on the lower surface of the membrane were fixed in methanol and then stained with 1% Crystal violet. The cells in at least five randomly selected fields were photographed under a microscope and counted. The data are presented as mean ± SD.

### Migration (wound-healing) assay

Migration assay was performed by wound-healing experiment by using the two-well culture-insert (Ibidi, Grafelfing, Bavaria, Germany). In brief, cells were cultured in the both wells of culture-insert. After stimulation, the culture-inserts were removed to allow cell migration. After 24 h, the migration levels were photographed in the microscope and images were quantified.

### siRNA transfection

Commercial siRNAs targeting human Smad1 and Smad5 were used for transfection experiments (ThermoFisher Scientific, Waltham, MA, USA). Cells at ~80% confluence were transfected with the indicated 20 nM siRNA (si-control, si-Smad1 or si-Smad5) using the Lipofactamine RNAiMAX Transfection Reagent (ThermoFisher Scientific, Waltham, MA, USA). Scrambled sequence siRNAs were used as negative controls.

### Statistical analysis

Data in the figures and text are representative of three independent experiments and are expressed as the mean ± SD. Statistical analyses were performed using the SPSS statistical software package (SPSS/PC+, SPSS Inc., Chicago, IL, USA). Inter-group comparisons of continuous variables were analyzed with Student's ttest or analysis of variance (ANOVA). A two-tailed *P*-value of < 0.05 was considered statistically significant.

## Results

### DAPT and RO4929097, γ-secretase activity inhibitors, inhibits E-cadherin expression in LN18 and LN229 glioma cells

**Figure 1:**
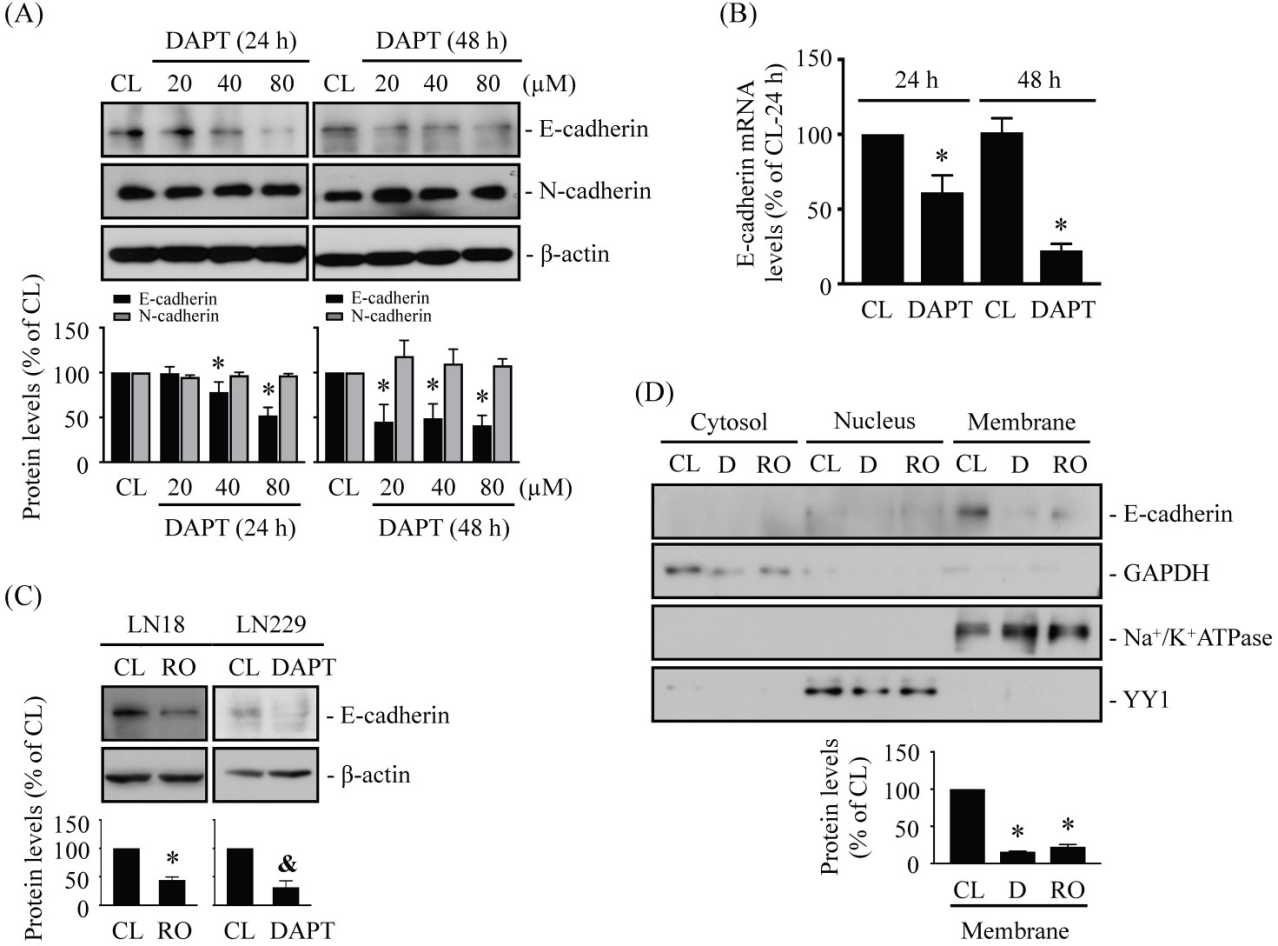
** DAPT and RO4929097, γ-secretase activity inhibitors, inhibits E-cadherin expression in LN18 and LN229 glioma cells.** (*A-B*) LN18 glioma cells were kept as controls (CL) or treated with DAPT (20, 40, or 80 µM) for 24 h or 48 h and then the protein (*A*) and/or mRNA (*B*) levels of N-/E-cadherin were examined by western blot and real-time PCR, respectively. (*C-D*) LN18 (*C-left panels and D*) and LN229 (*C-right panels*) glioma cells were kept as controls (CL) or treated with 1 µM RO4929097 (RO) or 80 µM DAPT (D) for 48 h and then the E-cadherin protein levels (*C*) and cellular localization (*D*) were examined by western blot and cell fractionation, respectively. Results in (*A, C-D*) are representative of three independent experiments. Data in (*A-D*) are shown as mean ± SD from three independent experiments. *, *P* < 0.05 vs*.* LN18-untreated control (CL) cells. &, *P* < 0.05 *vs.* LN229-untreated control (CL) cells.

N-cadherin and E-cadherin play important role in cell junctions and cancer metastasis. It has been found that cleavage of N-/E-cadherin by γ-secretase may help initiate the migration of cancer cells 18, 21. We assessed whether blocking γ-secretase activity by specific inhibitors, i.e., DAPT and RO4929097, affects N-/E-cadherin levels in human glioma cells. LN18 glioma cells were kept as controls or treated with DAPT (20, 40, or 80 μM) for 24 h and 48 h and then N-/E-cadherin protein and mRNA levels were examined. It was shown that DAPT causes downregulations of E-cadherin protein (Fig. 1A) and mRNA (Fig. 1B) levels in LN18 glioma cells in the dose- and time-dependent manners. However, DAPT did not affect N-cadherin protein levels (Fig. 1A). LN18 glioma cells treated with 1 μM RO4929097 (Fig. 1C, left panels) or LN229 glioma cells treated with 80 μM DAPT (Fig. 1C, right panels) showed similar inhibitory effects on E-cadherin protein expressions. Moreover, the results from protein localization assay showed that membrane E-cadherin protein level could be significantly decreased in LN18 glioma cells treated with both types of γ-secretase inhibitors (Fig. 1D).

### DAPT and RO4929097 promotes LN18 and LN229 glioma cell migration

**Figure 2:**
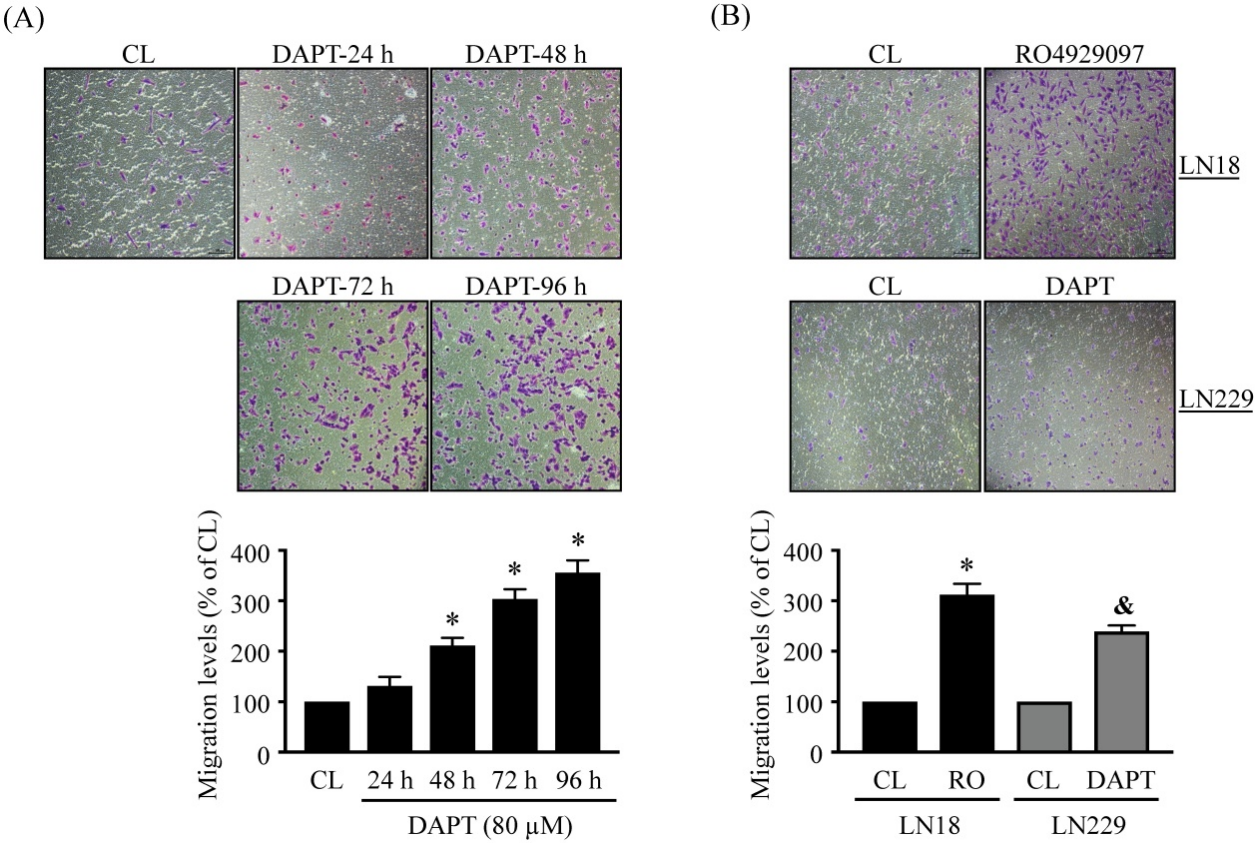
** DAPT and RO4929097 promotes LN18 and LN229 glioma cell migration by chemotaxis method.** (*A*) LN18 glioma cells were kept as controls (CL) or treated with 80 µM DAPT for 24, 48, 72, or 96 h and (*B*) LN18 (*upper panels*) and LN229 (*down panels*) glioma cells were kept as controls (CL) or treated with 1 µM RO4929097 (RO) or 80 µM DAPT for 96 h and cell migration were examined by chemotaxis method (transwell). Data are shown as mean ± SD from three independent experiments. *, *P* < 0.05 vs*.* LN18-untreated control (CL) cells. &, *P* < 0.05 *vs.* LN229-untreated control (CL) cells.

**Figure 3:**
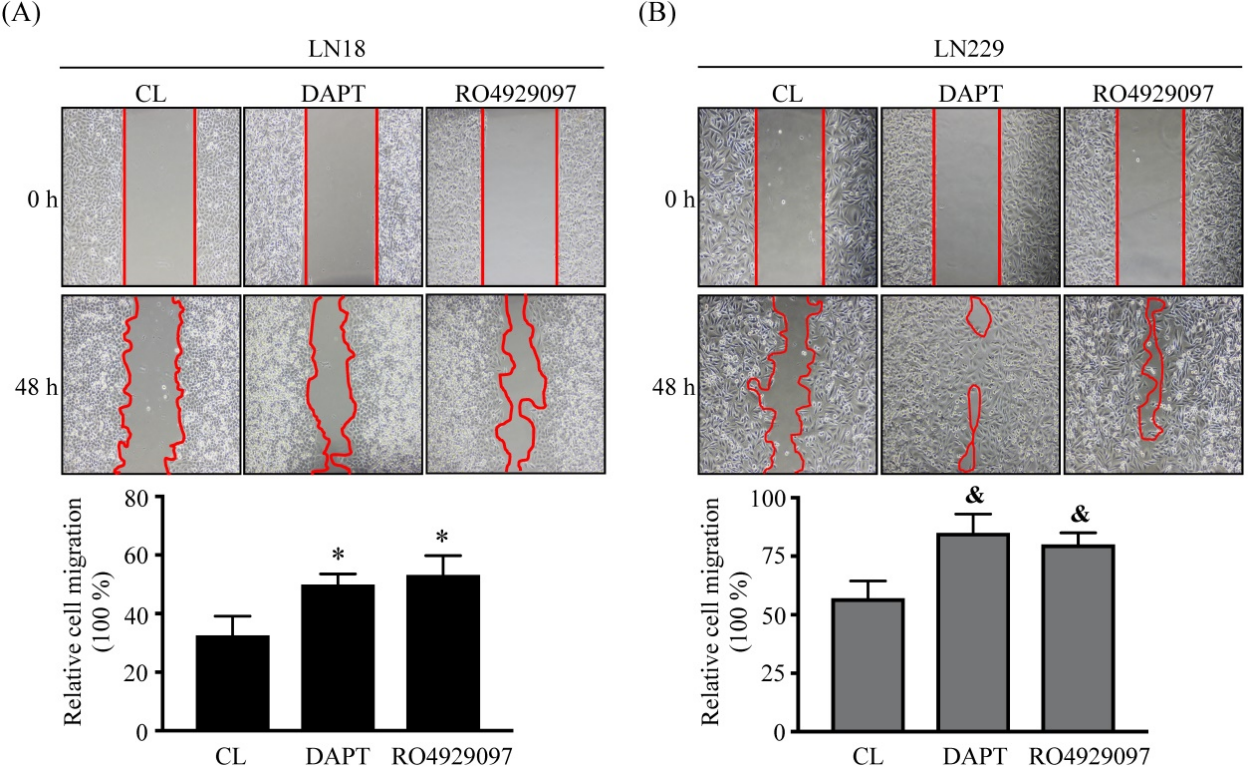
** DAPT and RO4929097 promotes LN18 and LN229 glioma cell migration by wound-healing method.** (*A-B*) LN18 (*A*) and LN229 (*B*) glioma cells were kept as controls (CL) or treated with 80 µM DAPT or 1 µM RO4929097 for 48 h and cell migration were examined by wound-healing method. Data are shown as mean ± SD from three independent experiments. *, *P* < 0.05 vs*.* LN18-untreated control (CL) cells. &, *P* < 0.05 *vs.* LN229-untreated control (CL) cells.

The migration levels of glioma cells were examined by using chemotaxis and wound-healing methods. LN18 glioma cells were kept as controls (CL) or treated with 80 μM DAPT for 24 h, 48 h, 72 h, and 96 h and then cell migration was examined by chemotaxis method. LN18 glioma cells treated with DAPT resulted in a significant increase in cell migration in a time-dependent manner (Fig. 2A). The numbers of migrated cells reached to a maximal level after 96 h of treatment compared to the untreated control cells, which were ~3 folds of untreated control cells. Both LN18 and LN229 glioma cells were kept as controls or treated with 80 μM DAPT or 1 μM RO4929097 for 48 or 96 h and then cell migration was examined by chemotaxis (Fig. 2B) and wound-healing (Fig. 3) methods. It was shown that both γ-secretase inhibitors, i.e., DAPT and RO4929097, have similar promoted effect on cell migration levels of LN18 (upper panels in Fig. 2B and Fig. 3A) and LN229 (down panels in Fig. 2B and Fig. 3B) glioma cells.

### DAPT-inhibited E-cadherin expression is regulated by BMP-independent Smad5 activation

**Figure 4:**
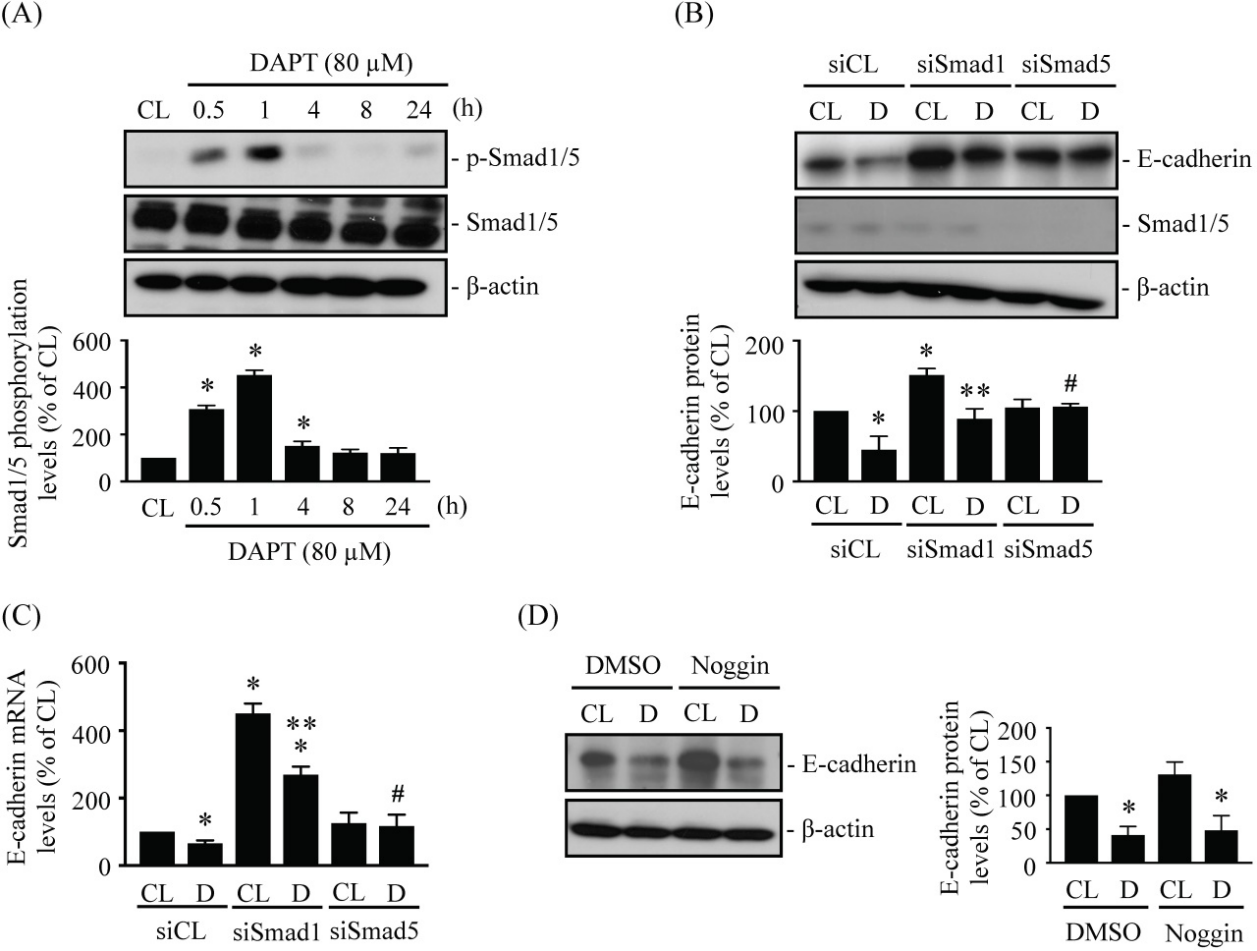
** DAPT-inhibited E-cadherin expression is regulated by BMP-independent Smad5 activation.** (*A*) LN18 glioma cells were kept as controls (CL) or treated with 80 µM DAPT for 0.5 h, 1 h, 4 h, 8 h, or 24 h and then Smad1/5 phosphorylation was examined by western blot. *(B-C)* LN18 glioma cells were pretreated with control (siCL), Smad1 (siSmad1)-, or Smad5 (siSmad5)-specific siRNA and then were kept as controls (CL) or treated with 80 µM DAPT (D) for 48 h. (*B*) E-cadherin/Smad1/5 protein and (*C*) E-cadherin mRNA levels were examined by western blot and real-time PCR, respectively. (*D*) LN18 glioma cells were pretreated with DMSO or Noggin (BMPs-specific antagonist) for 1 h and then were kept as controls (CL) or treated with 80 μM DAPT (D) for 48 h. The E-cadherin expression was examined by Western blot. Results in (*A-B, D*) are representative of three independent experiments. Data in (*A-D*) represent mean ± SD from three independent experiments. *, *P* < 0.05 *vs.* untreated control (CL) cells (*A*), siCL-CL-treated cells *(B-C)*, or DMSO-CL-treated cells *(D)*. **, *P* < 0.05 *vs.* siSmad1-CL-treated cells *(B-C)*. #, *P* < 0.05 *vs.* siCL-DAPT (D)-treated cells *(B-C)*.

BMP and Smad1/5 signaling may play important roles in glioma development 22, 25-26. LN18 glioma cells were kept as controls or treated with 80 μM DAPT for 0.5 h, 1 h, 4 h, 8 h, or 24 h and then Smad1/5 phosphorylation were examined. Treating cells with DAPT increased Smad1/5 phosphorylation within 0.5 h of treatment in LN18 glioma cells, with phosphorylation peaking within 1 h and returning to basal levels after 4 h of treatment (Fig. 4A). LN18 glioma cells were pretreated with control (siCL), Smad1 (siSmad1)-, or Smad5 (siSmad5)-specific siRNAs and then were kept as controls or treated with 80 μM DAPT for 48 h. Treatment with either Smad1- or Smad5-specific siRNAs resulted in ~50-80% reductions in target protein expression levels. The knockdown of Smad5 prevented DAPT-dependent repression of E-cadherin protein (Fig. 4B) and mRNA (Fig. 4C) levels in LN18 glioma cells compared to cells co-treated with control siRNA and DAPT. In addition, cells pretreated with Smad1-specific siRNA increased the basal endoenous E-cadherin protein (Fig. 4B) and mRNA (Fig. 4C) expressions in DAPT-untreated cells, and the DAPT treatment also further downregulated E-cadherin expressions in LN18 glioma cells (Fig. 4B and 4C). Smad1/5 are involved in signaling pathways downstream of BMPs; therefore, we examined whether the effect of DAPT on Smad5-E-cadherin is regulated by BMPs autocrine stimulation. Cells were pretreated with either DMSO or Noggin, a BMP-specific antagonist, for 1 h and then were kept as controls or treated with 80 μM DAPT for 48 h. It was shown that Noggin does not affect the inhibition of E-cadherin expression by DAPT in LN18 glioma cells (Fig. 4D).

### Smad5 signaling is involved in DAPT-promoted LN18 glioma cell migration

**Figure 5:**
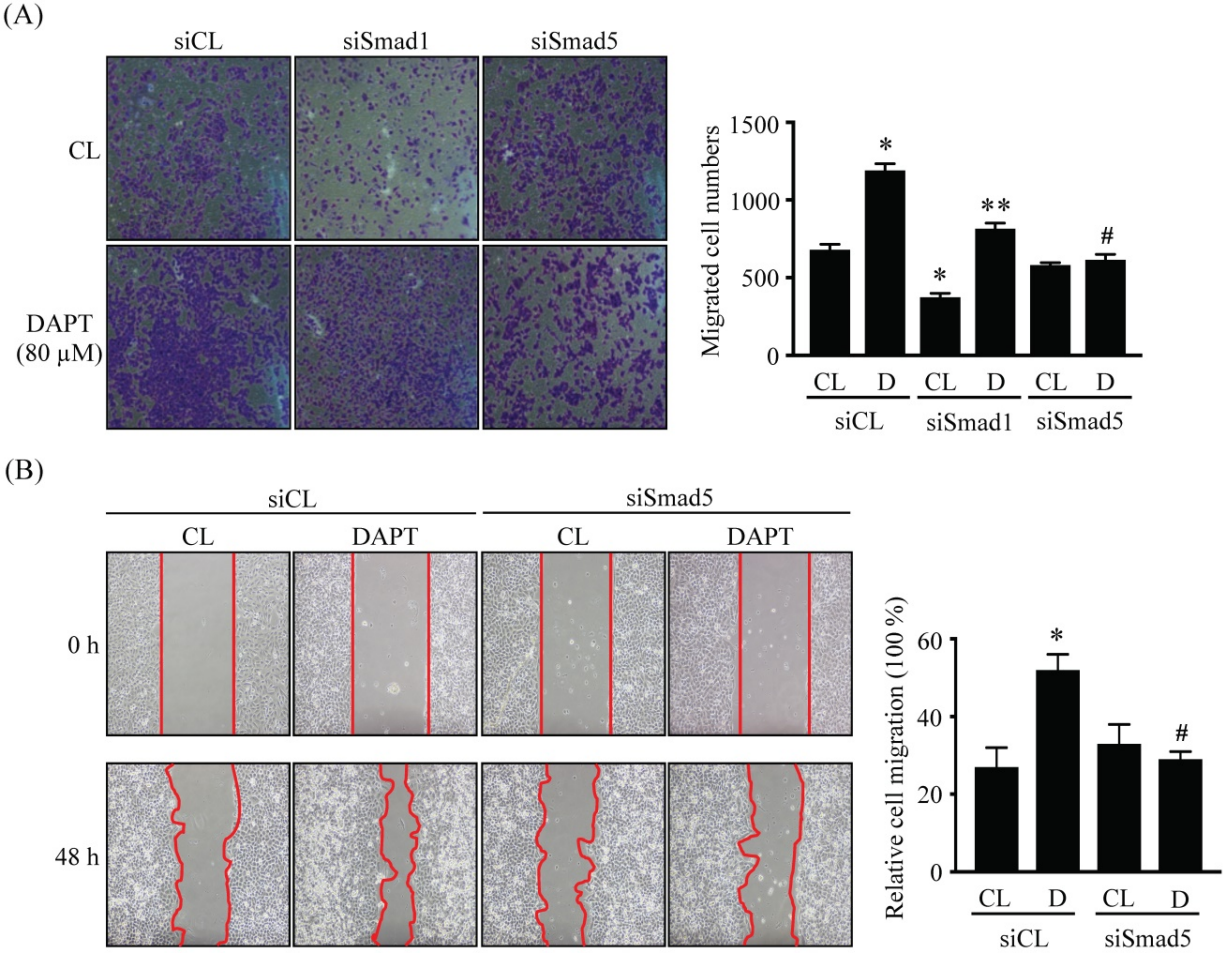
** Smad5 signaling is involved in DAPT-promoted LN18 glioma cell migration.** (*A-B*) LN18 glioma cells were pretreated with control (siCL)-, Smad1 (siSmad1)- or Smad5 (siSmad5)-specific siRNA and then were kept as controls (CL) or treated with 80 μM DAPT (D) for 48 h (wound-healing) or 96 h (chemotaxis). Cell migration was examined by chemotaxis (transwell) (*A*) and wound-healing (*B*) methods. Data in (*A-B*) are mean ± SEM from three independent experiments. *, *P* < 0.05 *vs.* siCL-CL-treated cells. **, *P* < 0.05 *vs.* siSmad1-CL-treated cells. #, *P* < 0.05 *vs.* siCL-DAPT (D)-treated cells.

LN18 glioma cells were pretreated with control (siCL), Smad1 (siSmad1)-, or Smad5 (siSmad5)-specific siRNA and then were kept as controls or treated with 80 μM DAPT for 48 or 96 h and then cell migration was examined by chemotaxis (Fig. 5A) and wound-healing (Fig. 5B) methods. It was shown that the gene knockdown of Smad5 significantly recovered the DAPT-promoted LN18 glioma cell migration compared to the cells-cotreating with control-specific siRNA and DAPT (Fig. 5A and 5B). Cells pretreated with Smad1-specific siRNA decreased the basal migration level in DAPT-untreated cells, and the DAPT treatment also further promoted LN18 glioma cell migration (Fig. 5A).

### Endogenous E-cadherin level affects LN18 and LN229 glioma cell migration

**Figure 6:**
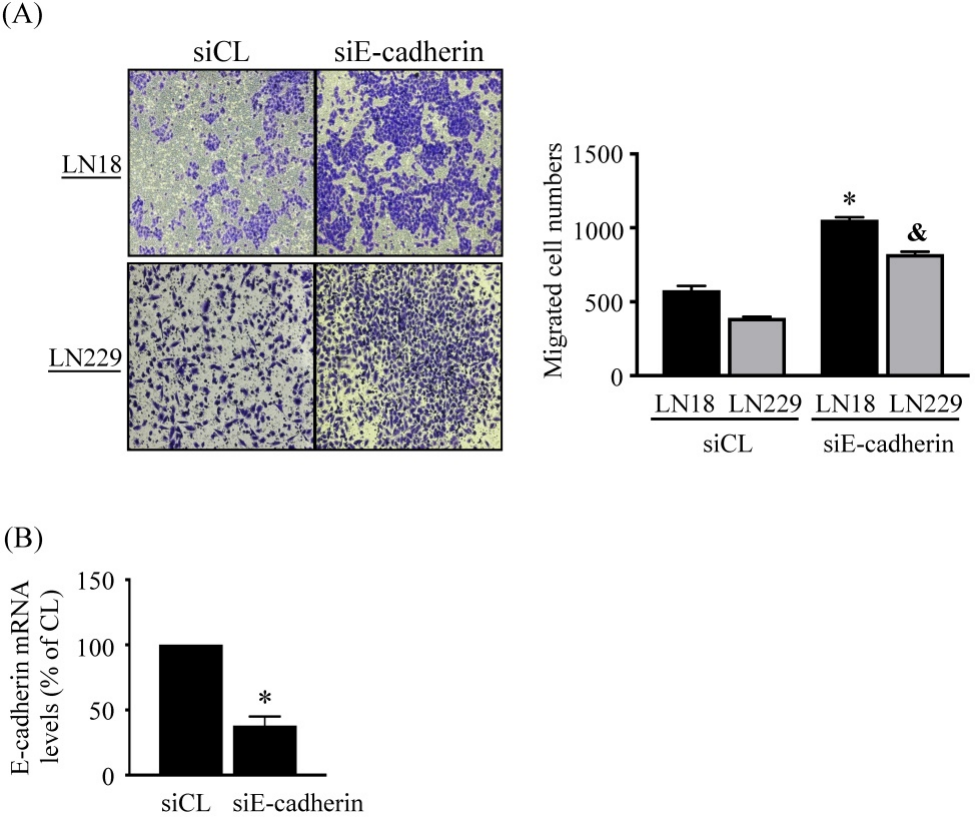
** Endogenous E-cadherin level affects LN18 and LN229 glioma cell migration.** (*A-B*) LN18 and LN229 glioma cells were treated with control (siCL) or E-cadherin (siE-cadherin)-specific siRNA for 96 h and then cell migration and endogenos E-cadherin mRNA expression were examined by chemotaxis methods and real-time PCR, respectively. Data in (*A-B*) are mean ± SEM from three independent experiments. *, *P* < 0.05 *vs.* LN18-siCL-treated cells. &, *P* < 0.05 *vs.* LN229-siCL-treated cells.

Finally, we determine whether endogenous E-cadherin affects glioma cell migration. LN18 and LN229 glioma cells were treated with control (siCL) or E-cadherin (siE-cadherin)-specific siRNA for 96 h and then cell migration was examined by chemotaxis methods. It was shown that the gene knockdown of E-cadherin significantly increases the migration levels of both types of cells compared to the cells-treating with control-specific siRNA (Fig. 6A). Cells treated with E-cadherin-specific siRNA resulted in ~60% reductions in target gene expression level (Fig. 6B).

## Discussion

**Figure 7:**
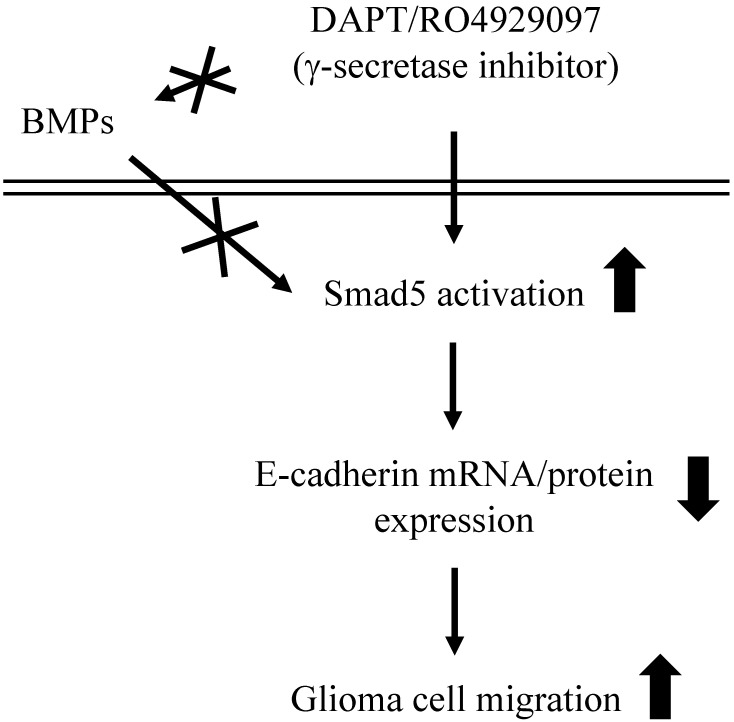
Schematic representation of signaling pathways regulating DAPT/RO4929097-promoted cell migration in human LN18/LN229 glioma cells.

This study has surprisingly revealed an unexpected role of γ-secretase activity inhibition by DAPT and RO4929097 in promoting glioma cell migration (summarized in Fig. 7). The systematic experiments demonstrated that (*i*) DAPT and RO4929097 downregulate E-cadherin (not N-cadherin) protein and mRNA expressions and subsequently promotes LN18 and LN229 glioma cells migration. (*ii*) This DAPT/RO4929097 effect on glioma cells is regulated by BMPs-independent Smad5 (not Smad1) activation. (*iii*) endogenous Smad1 plays a critical role in E-cadherin expression and consequent migration of glioma cells. Our findings provide new insights into the potential migration-promoting role of γ-secretase activity inhibition in malignant glioma.

E-cadherin, a critical adherens junction protein, is a well-established γ-secretase substrate 11. The cleavage of E-cadherin by γ-secretase induces disassembly of the adherens junction complex (made up of E-cadherin and β-catenin), resulting in intercellular junction disruption and the release of soluble β-catenin into the cytosol and nucleus to regulate Wnt signaling 12, 18. β-catenin released from γ-secretase cleavage of E-cadherin junction complexes in bone marrow stromal cells enhances osteoinduction during bone healing 30. In contrast, intercellular junction disruption by γ-secretase cleavage of E-cadherin is associated with metastasis and malignance of cancers, especially for breast cancer 18, 31-32. Because of this association, it has proposed that blocking γ-secretase activity in breast cancer cells should be tested in pharmacological studies. In our study, DAPT and RO4929097, two commonly used γ-secretase inhibitors, promoted glioma cell migration. Moreover, we found that the effects of DAPT/RO4929097 are mediated by the repression of E-cadherin mRNA and protein levels. This finding is surprising. In glioma cells, γ-secretase has been indicated as a potential therapeutic target because its enzymatic cleavage on another substrates, i.e., p75 and Notch, has been associated with glioma metastasis 15-16, 20. However, our study suggested another possible of DAPT effect on glioma cell migration, which is through E-cadherin expression regulation. Recently, the epithelial-to-mesenchymal transition (EMT) and shifts in E- and N-cadherin expression have been linked to glioma metastasis 33-35. Additionally, in clinical cases, N-cadherin expression appears to positively correlate with increasing grades of malignant gliomas 33. However, in contrast to N-cadherin, E-cadherin expression is low and its exact role in glioma cells is still unclear. Considering the information available, we proposed that although the level of E-cadherin in glioma cells is lower than that of N-cadherin, E-cadherin helps promote glioma cell migration. We also propose that although the effect of DAPT/RO4929097 on E-cadherin in glioma cells is through regulation of protein/mRNA expression levels, it is possible that DAPT/RO4929097 also affects other γ-secretase substrates to mediate this effect on E-cadherin. Finally, it is still unclear whether the high heterogeneity of glioma cells present leads to different outcomes upon γ-secretase activity inhibition. All of these questions should be further investigated to provide improved mechanistic explanations underlying glioma cell function.

It has recently been indicated that BMPs likely have a regulatory role in gliomas and thus have potential therapeutic capabilities. For example, BMP4 is able to promote differentiation, inhibit proliferation, and decrease tumorigenicity of glioma stem cells 25, 36. Additionally, BMP7 can suppress the tumorigenicity of stem-like glioblastoma cells 37. However, some studies have demonstrated positive correlations between BMP expression and the malignant grade of gliomas 38-39. Our present study indicates that the repression of E-cadherin mRNA and protein expression via DAPT treatment in glioma cells is regulated by BMP-independent Smad5 activation. We can exclude the possibility of autocrine BMP effects on DAPT-treated glioma cells because the BMP antagonist Noggin did not affect the repression of E-cadherin expression through Smad5 by DAPT treatment. Moreover, our findings indicate that although Smad1 is not involved in regulating DAPT inhibition of E-cadherin expression, basal endogenous Smad1 levels can regulate E-cadherin expression and subsequent glioma cell migration. This suggests that endogenous Smad1 may have a regulatory role in glioma cells. Although Smad1/5 signaling is a well-known downstream pathway from BMPs, accumulating evidence suggests that Smad1/5 signaling can also be activated independently of BMPs. For example, TGFβ and mechanical forces may also induce Smad1/5 phosphorylation without BMP stimulation 40-41. Further research is therefore needed to further examine whether DAPT affects the processing of other γ-secretase substrates and modulation of Smad1/5 signaling.

Our present study showed that Smad1/5 phosphorylation in LN18 glioma cells only persists for ~4 h after DAPT stimulation. But, the DAPT-elicited E-cadherin downregulation and subsequent glioma cell migration could persist for more than 24-96 h. Although the results from Smad1/5 gene knockdown experiments already confirmed the importeant role of Smad1/5 in DAPT effect on E-cadherin expression and glioma cell migration, the other signaling regulators, including others γ-secretase's substrates, mediating this time gap should be further elucidated in future. Moreover, the present results showed that only Smad5 could regulate E-cadherin expression in DAPT-treated glioma cells. And, although endogenous Smad1 itself could also affect E-cadherin expression, it shouldn't be the downstream mediator of DAPT. Smad1 and Smad5 have been found to share high amino acid identity and structure similarity. Most studies have demonstrated their similar activities and possible compensative mechanisms in regulating cell functions. However, in constrast, Smad1 and Smad5 have also been indicated to probably contribute to the diverse roles in the embryogenesis and hematopoiesis 42-43. It has been further elucidated that Smad1/5 activity might be affected and controlled by the different ligand stimulations and different membrane receptors and/or intracellular signaling proteins associations 44. Thus, these findings might support our results about distinct roles of Smad1 and Smad5 in DAPT-treated glioma cells, but their exact mechanisms still need more clear examination.

## Conclusion

This study reported that γ-secretase activity inhibition by DAPT/RO4929097 in LN18 and LN229 glioma cells promotes glioma cell migration by inhibiting E-cadherin mRNA and protein levels through BMP-independent Smad5 activation. These results provide new insights into the complex role of γ-secretase and its substrates in glioma metastasis regulation. Moreover, although E-cadherin levels in glioma cells are extremely low, our results demonstrate an important role for E-cadherins in regulating malignant gliomas. Additionally, we found that endogenous Smad1 may have a regulatory role in glioma cells. Our present study thus indicates the need for further elucidation of pathways related to glioma cell development and future clinical developments.

## Abbreviations

BMP: bone morphogenetic protein
DAPT: N-[N-(3,5-difluorophenacetyl)-L-alanyl]-S-phenylglycine t-butyl ester
EMT: epithelial-to-mesenchymal transition
